# A robust algorithm for authenticated health data access via blockchain and cloud computing

**DOI:** 10.1371/journal.pone.0307039

**Published:** 2024-09-23

**Authors:** Ali Shahzad, Wenyu Chen, Momina Shaheen, Yin Zhang, Faizan Ahmad

**Affiliations:** 1 School of Computer Science and Engineering, University of Electronic Science and Technology of China, Chengdu, Sichuan, China; 2 Department of Computing, University of Roehampton London, London, United Kingdom; 3 School of Information and Communication Engineering, University of Electronic Science and Technology of China, Chengdu, Sichuan, China; 4 Cardiff School of Technologies, Cardiff Metropolitan University, Cardiff, United Kingdom; Jazan University, SAUDI ARABIA

## Abstract

In modern healthcare, providers increasingly use cloud services to store and share electronic medical records. However, traditional cloud hosting, which depends on intermediaries, poses risks to privacy and security, including inadequate control over access, data auditing, and tracking data origins. Additionally, current schemes face significant limitations such as scalability concerns, high computational overhead, practical implementation challenges, and issues with interoperability and data standardization. Unauthorized data access by cloud providers further exacerbates these concerns. Blockchain technology, known for its secure and decentralized nature, offers a solution by enabling secure data auditing in sharing systems. This research integrates blockchain into healthcare for efficient record management. We proposed a blockchain-based method for secure EHR management and integrated Ciphertext-Policy Attribute-Based Encryption (CP-ABE) for fine-grained access control. The proposed algorithm combines blockchain and smart contracts with a cloud-based healthcare Service Management System (SMS) to ensure secure and accessible EHRs. Smart contracts automate key management, encryption, and decryption processes, enhancing data security and integrity. The blockchain ledger authenticates data transactions, while the cloud provides scalability. The SMS manages access requests, enhancing resource allocation and response times. A dual authentication system confirms patient keys before granting data access, with failed attempts leading to access revocation and incident logging. Our analyses show that this algorithm significantly improves the security and efficiency of health data exchanges. By combining blockchain’s decentralized structure with the cloud’s scalability, this approach significantly improves EHR security protocols in modern healthcare setting.

## 1 Introduction

Since the early 21st century, significant advancements in digital technologies have revolutionized the global healthcare sector. This revolution is most notable in the transition from traditional paper records to digital ones, specifically Electronic Health Records (EHRs) [1, 2]. EHRs, structured digital repositories, store various patient data like lab results, medical history, and medication records [3]. They offer several benefits over paper records, such as reduced labor, time, and space requirements [4].

The healthcare industry, with its growing reliance on various digital applications and services, generates immense amounts of data daily. This increase in data has made robust storage and management services essential, with cloud computing emerging as a key solution [5]. Cloud Service Providers (CSPs) offer cost-effective and efficient infrastructure for data storage and processing, attracting both individual and institutional users [6, 7]. Cloud-based electronic health (eHealth) systems efficiently manage EHRs, allowing for secure data sharing across healthcare networks [8, 9].

However, the use of cloud-based eHealth systems raises significant security and privacy concerns [10]. When EHRs are stored on cloud servers, issues such as data ownership, access control, and security breaches become critical challenges. These challenges can hinder the adoption and growth of cloud-based services [11, 12].

One effective way for data security within cloud computing, encryption stands as a best technology among them. Researchers often turn to encryption technologies to secure data that is outsourced to the cloud. Among these technologies, ciphertext-policy attribute-based encryption is recognized as an effective way to implement access control on less reliable cloud storage servers. Despite the existence of numerous schemes aimed at ensuring data confidentiality and user privacy, challenges persist, particularly in the effective identification, tracking, and revocation of permissions for users who misuse their access, underscoring the need for enhanced access control systems. These systems must not only provide ease of access for authorized users but must also have stringent measures for promptly revoking access when a security breach occurs. Additionally, incorporating blockchain technology can enhance security. Blockchain’s transparent and immutable record-keeping is useful for secure and efficient data management, addressing concerns about data integrity [13–15].

Therefore, we propose a blockchain-enhanced security model for EMR, emphasizing traceable and direct access revocation. our research provides a scalable, efficient, and practical solution for secure healthcare data management. By combining blockchain’s decentralized structure with the scalability of cloud computing, our approach significantly improves EHR security protocols, ensuring robust data security and seamless performance in modern healthcare settings.

## Our key contributions and innovations

1. **Blockchain-Empowered Security**: Our research introduces a blockchain-based method to protect privacy and secure Electronic Medical Records (EMRs). This approach leverages smart contracts to automate key management, encryption, and decryption processes, ensuring robust security and flexible access revocation.
2. **Advanced Encryption with CP-ABE**: We integrate Ciphertext-Policy Attribute-Based Encryption (CP-ABE) to enhance data security by embedding access control policies within the ciphertext. This ensures fine-grained access control, allowing only authorized personnel to access sensitive medical data. Additionally, the dual authentication system confirms patient keys before granting data access, with failed attempts leading to access revocation and incident logging.
3. **Comprehensive Security Analysis**: We performed an in-depth security analysis demonstrating the scheme’s resilience against various cyber threats and its efficiency in key management, encryption, and decryption processes.
4. **Performance Analysis and Scalability**: We conducted extensive performance evaluation, including detailed simulation experiments. Our results show that the scheme performs well in terms of communication and storage efficiency, handling large data volumes effectively. The assessment within a blockchain environment, focusing on throughput and delay, confirmed its effectiveness and practical applicability, making it highly suitable for modern healthcare systems.

The paper is organized as follows: Section 2 presents the related works, Section 4 details the system architecture, Section 7 covers the security model, Sections 9 and 10 present the security analysis and protocol design, while Section 11 presents simulation and experiments, and Section 14 summarizes our findings.

## 2 Related work

In the domain of blockchain technology for access control schemes with traceability and revocability for healthcare data management, a spectrum of research has been conducted, each contributing uniquely to the field.

T. Benil and J. Jasper’s work, investigates the utilization of blockchain for managing electronic health records (EHRs), focusing on crucial aspects of data integrity and access control. However, this study brings to light concerns regarding the scalability and computational efficiency of blockchain technologies in healthcare contexts [16]. Similarly, K. Anil and Dr. Megha Kamble’s “Health Block” presents a blockchain-based system adapted for healthcare data, prioritizing user-friendliness and data integrity. Aside from its innovative approach, the study falls short in thoroughly analyzing the scalability and transaction costs associated with such systems [17]. Maria Guzman Lizama and her team’s systematic review of the application of blockchain for medical image sharing in the cloud environment emphasizes the security benefits of this technology. But, the review underscores the necessity for further research focusing on the scalability and efficiency of blockchain in medical contexts [18].

In Integration of Blockchain and Cloud Computing in Telemedicine and Healthcare by Asma Albassam et al. offer an insightful overview of the integration potential of blockchain and cloud computing. The paper identifies an urgent need for additional research in data manipulation and secure storage patterns within these systems [19]. Shujiang Xu and her colleagues introduce a blockchain-based scheme enhancing privacy in mobile health. Despite its advancements, the research highlights a gap in understanding its scalability and practical applicability in real-world scenarios [20].

Mercy Ehiwuogwu’s investigation into the integration of blockchain technology in EHR systems is a notable contribution that discusses the enhancement of security and privacy. However, this study does not examine deeply the practical implementation challenges that such systems might face [21]. The work of Insaf Boumezbeur and Karim Zarour on hybrid encryption for healthcare data sharing in cloud environments shows promising improvements in performance. Yet, it also acknowledges the necessity for more comprehensive research into the scalability and practicality of these systems [22].

The paper “Privacy and Security of Blockchain in Healthcare” [23] offers a thorough analysis of the diverse applications of blockchain in healthcare, highlighting enhanced data integrity and patient control over their data. However, the study calls for more case studies and real-world applications to demonstrate the practicality of these blockchain solutions in healthcare settings. [24] Upadrista addresses the potential of blockchain in remote health monitoring. It brings attention to significant challenges, including scalability, energy consumption, and the need for standardized health data formats. Anandarshan K Hebballi and his team focus on enhancing patient data privacy [25]. The study suggests a need for further exploration into the computational overhead and the practicality of implementing such systems in healthcare settings.

Xiaohong Zhang, Wenqi Du, and Ata Jahangir Moshayedi present a blockchain-based security model for data storage, introducing features like traceability and revocation. However, the study recognizes the need for more analysis of the scalability and applicability of these features in real-world scenarios [26]. Zhe Liu et al. [27] contribute to the field with a user revocation scheme in CP-ABE systems. However, his research calls for more exploration of practical implementation aspects of their scheme.

Hui Cui in “An Efficient and Expressive Ciphertext-Policy Attribute-Based Encryption Scheme” addresses the privacy challenges in ABE systems. The research indicates a gap in understanding the real-world application and system integration of these schemes [28]. Takeru Naruse and his team, in their paper “Attribute-Based Encryption with Attribute Revocation and Grant Function,” introduce a proxy re-encryption-based ABE scheme. The study highlights the need for scalability research, particularly in large-scale cloud environments [29].

Humera Aqeel and Syed Taqi Ali’s in [30] “Directly Revocable Attribute-Based Encryption Scheme under Ciphertext-Policy” offers an efficient user revocation method. However, the paper acknowledges the lack of in-depth exploration into scalability and computational overhead challenges.

Shangping Wang [31] in his paper proposes a traceable CP-ABE scheme for cloud storage, focusing on collusion resistance and computational efficiency. However,The main drawback is that even when a malicious user is traced, they cannot be efficiently revoked from the crypto system. The “Traceable-then-revocable ciphertext-policy attribute-based encryption scheme” paper develop a scheme with enhanced traceability and direct user revocation. However author mentioned that system can only achieve white-box traceability, which is less robust compared to black-box traceability [32].

Yong Cheng et al.’s in “Directly Revocable Attribute-Based Encryption” for CP-ABE systems in cryptographic cloud storage focuses on reducing the data owner’s workload. Still, it recognizes the potential increase in data publication and retrieval costs as a drawback [33].

Zaid Ameen Abduljabbar’s work emphasizes the need for secure and efficient key management protocols in healthcare settings, highlighting their proposed scheme’s resilience against various security threats [34]. Samir M. Umran et al. demonstrate the integration of blockchain with their work to ensure data security and privacy in industrial settings, highlighting challenges in scalability and computational efficiency [15].

Samir and his team discusses the application of blockchain to enhance the security and reliability of their work, pointing out the need for lightweight cryptographic solutions to address resource constraints [13]. On the other hand Umran and Songfeng emphasizes in their work the importance of efficient consensus algorithms and data encryption mechanisms to secure industrial IoT data while maintaining low power consumption and high performance [14].

Similarly Vincent Omollo introduces an elliptic curve cryptography-based protocol to ensure data integrity and confidentiality in healthcare communications, addressing key concerns related to scalability and practical implementation in real-world scenarios [35]. Mustafa A. Al Sibahee focuses on enhancing security through biometric validation techniques, ensuring robust protection against unauthorized access in healthcare devices [36].

Lastly Umran presented the application of blockchain in industrial settings, emphasizing the need for scalable and efficient solutions to manage and secure data [37].

Each of these studies contributes valuable insights into the evolving field of blockchain and encryption technologies in healthcare data management, highlighting both their potential and the challenges that need to be addressed.

Despite the valuable contributions of these studies, several key limitations and gaps can be identified:

1. **Scalability Concerns**: Many existing schemes face scalability challenges when applied to large-scale healthcare systems or environments with high data volumes and numerous users. Further research is needed to ensure the practical scalability of these solutions in real-world healthcare settings.
2. **Computational Overhead**: Some of the proposed schemes introduce significant computational overhead, which may hinder their performance and efficiency, particularly in resource-constrained environments or with time-sensitive healthcare applications.
3. **Practical Implementation Challenges**: While many studies provide theoretical frameworks or simulations, there is a lack of comprehensive guidance and practical implementations to facilitate the seamless integration of these solutions into existing healthcare information systems and workflows.
4. **Interoperability and Data Standardization**: The integration of blockchain technology and secure access control mechanisms with diverse electronic health record (EHR) systems and healthcare data formats remains a challenge, highlighting the need for standardization and interoperability efforts.
5. **User Acceptance and Adoption**: The successful adoption of these solutions in healthcare settings requires addressing user acceptance factors, such as ease of use, training, and change management strategies, which have received limited attention in existing research.

Our research aims to address these gaps by proposing a scalable, efficient, and practical blockchain-based solution for secure healthcare data management, with a focus on traceability, direct access revocation, and seamless integration with existing healthcare systems and workflows.

## 3 Preliminaries

In this section, we provide an overview of the essential concepts and technologies used in our research, which are crucial for understanding the security model and proof of our proposed scheme.

### 3.1 Blockchain technology

Blockchain technology forms the backbone of our secure data management system. It is a decentralized ledger that records transactions across multiple nodes to ensure data integrity, transparency, and immutability. Each block in the blockchain contains a cryptographic hash of the previous block, a timestamp, and transaction data, creating a secure and tamper-proof chain of records [38].

### 3.2 Cloud computing

Cloud computing offers scalable and cost-effective infrastructure for storing and processing large volumes of healthcare data. Cloud Service Providers (CSPs) enable healthcare organizations to store Electronic Health Records (EHRs) in a secure, remote environment, facilitating efficient data sharing and management across healthcare networks [39].

### 3.3 Ciphertext-Policy Attribute-Based Encryption (CP-ABE)

CP-ABE is an advanced encryption technique that enhances data security by embedding access control policies within the ciphertext. In CP-ABE, the data owner defines an access policy and encrypts the data such that only users whose attributes satisfy the policy can decrypt the information. This method ensures that only authorized personnel can access sensitive medical data [40].

### 3.4 Smart contracts

Smart contracts are self-executing contracts with the terms of the agreement directly written into code. They run on the blockchain, enabling automated and secure transactions without intermediaries. In our system, smart contracts automate key management, encryption, and decryption processes for EHRs, ensuring that only authorized access is granted [41].

### 3.5 Notation and symbols

This section introduces the important symbols, notations, and cryptographic assumptions used throughout the paper as shown in Table 1.

**Table 1:** Notation and symbols.

Symbol	Description
G, GT	Cyclic groups of prime order p; G is the source group, and GT is the target group.
g	Generator of the group G.
e	Bilinear map *e* : *G* × *G* → *GT*.
H	Hash function mapping binary strings to elements of G, *H* : {0, 1}* → *G*.
*α*, *β*, a, b	Randomly chosen values from the set *Z*_*p*_.
PK	Public key used in the encryption scheme.
MK	Master key kept secret and used in the key generation process.
ui	User identifier within the system.
Lui	Attribute list for user i.
SKui	Secret key for a user i.
C	Ciphertext produced by the encryption algorithm.
Pi	Access policy for data Mi.
Ti	Access tree corresponding to Pi.
s	Random element from *Z*_*p*_ used in the encryption process.
D	Decryption key for user ui.
λ	Lagrange coefficients used in polynomial interpolation.
f	Function to extract plaintext from decrypted messages.
addrID	Unique identifier for an account based on the user’s password.
AC	Authentication Center responsible for verifying and delegating decryption.
SMS	Service Management System handling administrative tasks such as verifying decryption keys and tracking malicious users.

### 3.6 Bilinear maps

A bilinear map *e* : *G*_1_ × *G*_1_ → *G*_*T*_ has the following properties:

1. **Bilinearity**: For all *u*, *v* ∈ *G*_1_ and $a,b\in Z_{p}$, *e*(*u*^*a*^, *v*^*b*^) = *e*(*u*, *v*)^*ab*^.
2. **Non-degeneracy**: There exists *g* ∈ *G*_1_ such that *e*(*g*, *g*) ≠ 1.
3. **Computability**: There is an efficient algorithm to compute *e*(*u*, *v*) for all *u*, *v* ∈ *G*_1_.

### 3.7 Decisional Bilinear Diffie-Hellman (DBDH) assumption

The DBDH assumption states that for any probabilistic polynomial-time adversary, the advantage in distinguishing between the tuple (*g*, *g*^*a*^, *g*^*b*^, *g*^*c*^, *e*(*g*, *g*)^*abc*^) and (*g*, *g*^*a*^, *g*^*b*^, *g*^*c*^, *T*) for a random *T* ∈ *G*_*T*_ is negligible.

### 3.8 Integration of technologies

The integration of blockchain, cloud computing, CP-ABE, and smart contracts creates a synergistic framework that addresses the key challenges of data security, access control, and scalability in healthcare. Blockchain ensures data integrity and provides a transparent audit trail, while cloud computing offers scalable storage solutions. CP-ABE enhances data security with fine-grained access control, and smart contracts automate and secure the data management processes. Together, these technologies form a comprehensive solution for managing EHRs, ensuring that patient data is secure, accessible, and efficiently managed across healthcare systems.

By combining these advanced technologies, our research proposes an approach to secure healthcare data management, addressing the critical concerns of data privacy, security, and efficiency in the modern healthcare landscape.

## 4 The system architecture of data access control

**Fig 1:**
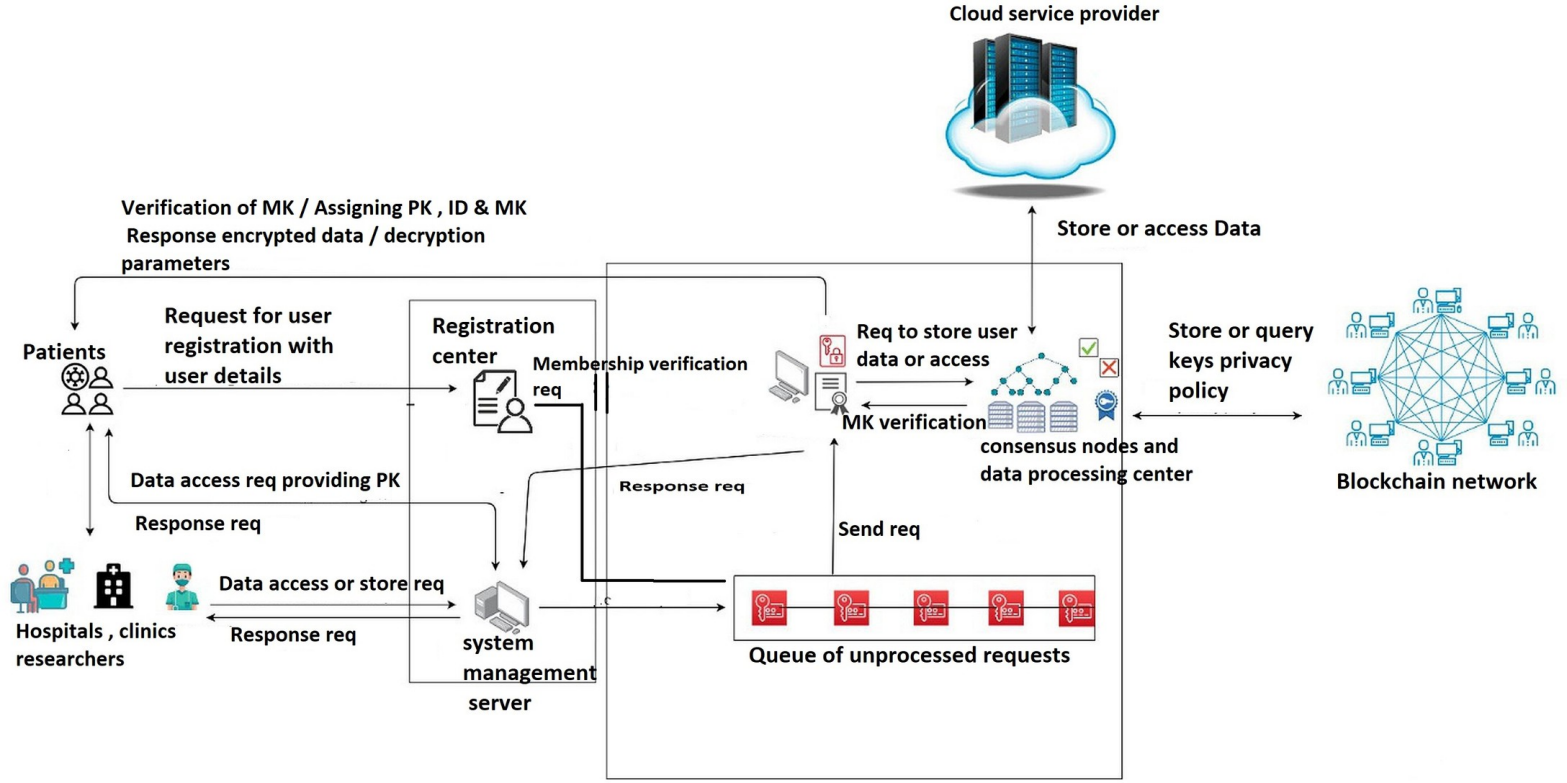
System design.

Our system design introduces a secure, privacy data exchange framework, detailed in the system architecture depicted in Fig 1. This framework comprises nine key components, each outlined below.

### 4.1 Patients

In our system, patients are main participants. By enrolling in the system, they become active blockchain users, each receiving a unique identifier (ID), a public key (PK), and a master key (MK). It’s crucial for patients to securely safe their MK.

### 4.2 Medical practitioners

Medical practitioners are enabled to request or upload data using Service management system (SMS), leveraging the patient’s PK. Data is stored on a cloud service provider (CSP) after the patient’s MK is authenticated.

### 4.3 Registration center

Registration centers get requests from patients and medical practitioners to join the network. Once the registration center gets details from users after verifying them, the registration center will generate ID, PK, and MK for the user. later, the registration center requests the Authentication center to verify these keys with the users.

### 4.4 Service management system (SMS)

The SMS serves as a reliable administrative entity, facilitating hospital services to users. It maintains a database of public keys and IDs for medical professionals, processes data access or storage requests, and oversees user activities. It has the authority to withdraw data access in case of suspicious activities.

### 4.5 Authentication center (AC)

The Authentication Center (AC) plays a crucial role in validating user identities, managing data storage and access on the Cloud Service Provider (CSP), and encrypting user data. Integrated with smart contracts, the AC automates and secures key management, encryption, decryption, and access control processes. Smart contracts within the AC ensure the generation, distribution, and management of cryptographic keys; enforce access policies during encryption; verify user attributes during decryption; and log all access attempts immutably on the blockchain. This combination enhances the system’s efficiency, security, and transparency, reducing the risk of human error and unauthorized access.

### 4.6 Processing and consensus nodes

These nodes manage data processing within the blockchain network. Data packages from the authentication center are encrypted and logged by processing nodes, then sent for validation. A majority consensus among these nodes is required for a block to be added to the blockchain. Failed verifications return the block to the original node.

### 4.7 Blockchain

The blockchain is providing unified identity verification. It maintains a database of user details, including IDs, PKs, MKs, and specific information for both patients and medical practitioners.

### 4.8 Queue of unprocessed requests

In this layer of the system, all the unprocessed requests will wait for their turn to be executed by the AC. From this, our system would not miss any requests.

### 4.9 Cloud service provider (CSP)

In our proposed system, the data storage layer is managed by a semi-trusted Cloud Service Provider (CSP). The CSP is responsible for storing all medical data, which has been encrypted by the Authentication Center (AC) prior to storage. This ensures that even within a semi-trusted environment, the data remains secure and inaccessible to unauthorized parties. The CSP offers scalable and reliable storage solutions, capable of efficiently handling large volumes of data. When access is requested, the AC verifies the user’s identity and attributes against the blockchain data. Upon successful verification, the AC retrieves the encrypted data from the CSP and provides the decryption parameters to the authorized user. This approach leverages the scalability and availability of cloud storage while maintaining stringent security protocols through encryption and blockchain-based logging.

## 5 System flow

### 5.1 User registration

In this system, first users will request to the registration center to be a member of our system by sending their details UD, and against it, the registration center will generate user ID, PK, and data access policy accordingly and request for MK to the AC. once the AC gets a request for MK from the registration center AC will verify with the user its identity and later AC will generate MK for the user and the user must store this MK secretly. Once the user gets its MK, the AC sends a request to processing and consensus nodes to create a block and store user details, user ID, data access policy, user PK, and MK into the blockchain.

### 5.2 Case 1: Data accessing and storing

When a patient visits a hospital and hospital wants to store or access Electronic Medical Records (EMRs) below steps will be taken.

**Fig 2:**
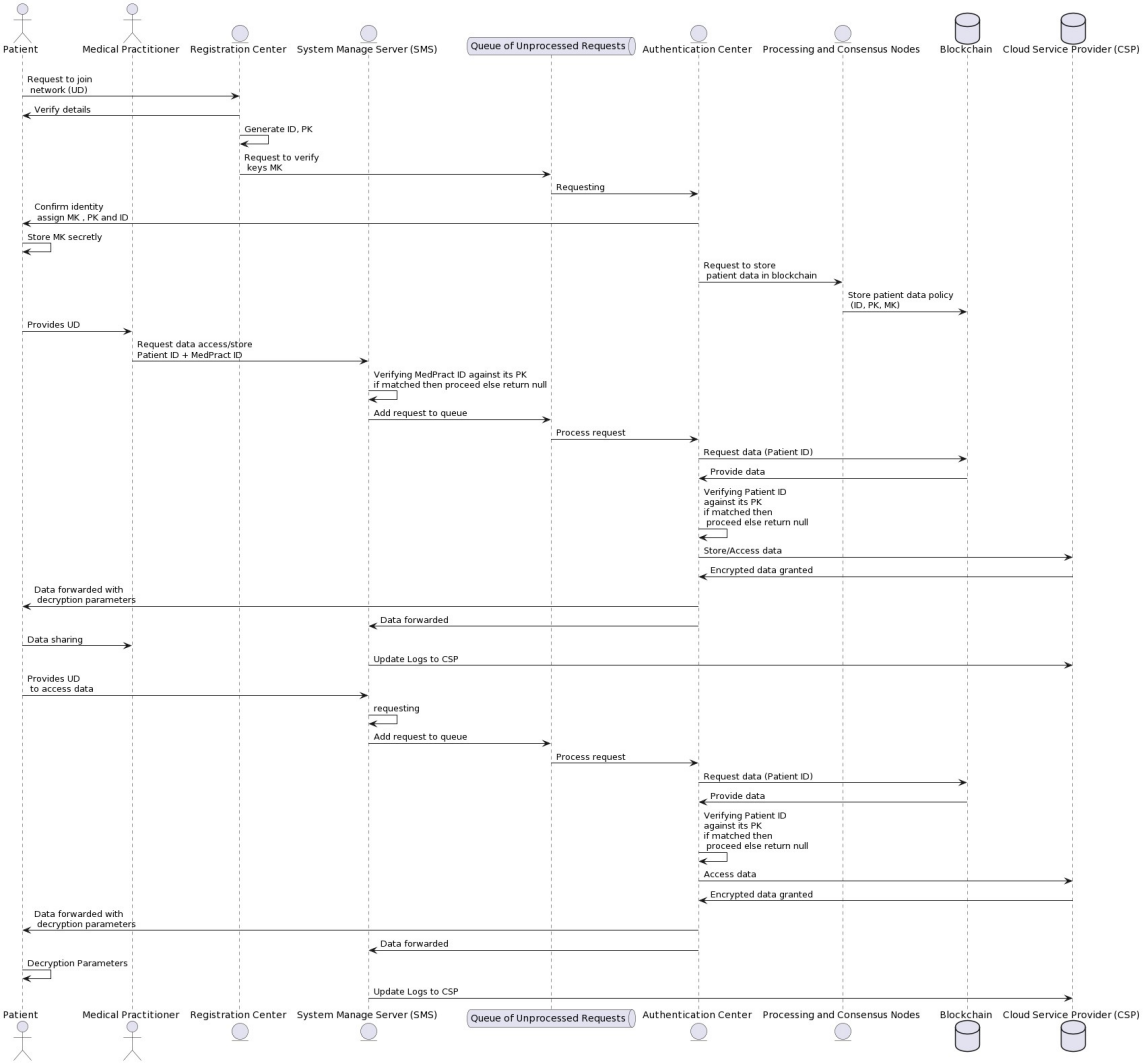
UML diagram of case 1 and case 2.

**Rules of Authentication and Data Retrieval**:

1. **Request Submission**: When hospital staff need to access a patient’s Electronic Medical Records (EMRs), they initiate the process by submitting their attributes to the Service management system (SMS) with Patients data. This is the first step in ensuring secure access to sensitive medical data.
2. **Request Queue**: Submitted requests undergo initial verification by the SMS, where the medical practitioner’s public key (Pk) is matched against their ID in local databases. Verified requests are then added to a queue of unprocessed requests, ensuring no request is missed.
3. **Authentication and Data Retrieval**:
  - **Forwarding to Authentication Center (AC)**: The queued requests are forwarded to the AC, where a smart contract verifies the user’s details and attributes against the blockchain data.
  - **Fetching Encrypted EMRs**: Upon successful authentication, the smart contract fetches the encrypted Electronic Medical Records (EMRs) from the Cloud Service Provider (CSP), as illustrated in Fig 2.
  - **Generating and Verifying Hashes**: Before storing or retrieving the data, a cryptographic hash of the data is generated using the hash function *H* : {0, 1}* → *G*. This hash is stored along with the encrypted data. When the data is retrieved, the hash is recalculated and compared to the stored hash to ensure the data has not been tampered with. This process ensures data integrity and authenticity, protecting the stored data from potential manipulation and unauthorized changes.
  - **Transmitting Decryption Parameters**: The smart contract transmits the decryption parameters to the authorized user, ensuring only authenticated users can access the sensitive data.
4. **Data Decryption Request**: Simultaneously, the AC sends intermediary parameters back to the SMS to monitor and control the decryption activities, enhancing overall data access security.
5. **Data Decryption and Access**: With the received decryption parameters and their private key, users can decrypt and access the EMRs, ensuring the confidentiality of patient records during access.
6. **Access Denial and Logging**: In the event of a mismatch or unauthorized access attempt, the SMS denies access and logs the incident. These logs, stored on the CSP, are crucial for tracking potential breaches and maintaining data security.
7. **Data Storage Process**:EMRs created and stored by the hospital are encrypted using Attribute-Based Encryption (ABE) by the AC after verification with the patient’s master key (MK). The encrypted records are then hashed, and both the encrypted data and its hash are securely stored on the CSP. This process ensures that any unauthorized changes to the data can be detected.

### 5.3 Case 2: Data accessing

When an individual patient sends a request to SMS for data access or storage.

**Rules of Authentication and Data Retrieval**:

1. **Request Submission**:
  - When a patient wants to access Electronic Medical Records (EMRs), the patient send a request to the Service management system (SMS) with its data
2. **Request Queue**: After a request submission, SMS transmits the request to a queue of unprocessed requests, awaiting its turn for further processing.
3. **Authentication and Data Retrieval**:
  - **Forwarding to Authentication Center (AC)**: The queued requests are forwarded to the AC, where a smart contract verifies the user’s details and attributes against the blockchain data.
  - **Fetching Encrypted EMRs**: Upon successful authentication, the smart contract fetches the encrypted Electronic Medical Records (EMRs) from the Cloud Service Provider (CSP).
  - **Verifying Hashes**: When the data is retrieved, the hash is recalculated and compared to the stored hash to ensure the data has not been tampered with.
  - **Transmitting Decryption Parameters**: The smart contract transmits the decryption parameters to the authorized user, ensuring only authenticated users can access the sensitive data.
4. **Data Decryption Request**: Simultaneously, the AC sends intermediary parameters back to the SMS to monitor and control the decryption activities, enhancing overall data access security.
5. **Data Decryption and Access**: With the received decryption parameters and their private key, users can decrypt and access the EMRs, ensuring the confidentiality of patient records during access.
6. **Access Denial and Logging**: In the event of a mismatch or unauthorized access attempt, the SMS denies access and logs the incident. These logs, stored on the CSP, are crucial for tracking potential breaches and maintaining data security.

## 6 Protocol illustration

We employ a robust and secure encryption algorithm, Ciphertext-Policy Attribute-Based Encryption (CP-ABE), integrated with blockchain technology, to ensure the confidentiality, integrity, and traceability of Electronic Health Records (EHRs). The Security Algorithm Framework consists of cryptographic operations for secure and traceable EHR management in cloud systems (as shown in Table 2). Our implementation includes the following key components:

**Table 2:** Security algorithm framework.

1: **procedure** SystemSetup(λ)
2: Select distinct groups *G* and *G*_*T*_ with a prime order *p*, and choose an element *g* from *G*.
3: Select a bilinear map function *e* that maps elements from *G* *G to *G*_*T*_.
4: Randomly choose values *α* and *β* from the set $Z_{p}$. Subsequently, calculate *g*^*α*^, *g*^*β*^, and *e*(*g*, *g*)^*αβ*^.
5: Define a hash function *H* that converts binary strings *H* : {0, 1}* → *G*
6: **return** (*g*, *g*^*α*^, *g*^*β*^, *e*(*g*, *g*)^*αβ*^, *H*)
7: **end procedure**
8: **procedure** KeyGeneration(*g*, *g*^*α*^, *g*^*β*^)
9: For user *i* with attributes *A*_*i*_, pick random $r_{i}\in Z_{p}$
10: Compute secret keys *SK*_*i*_ for each attribute *a* ∈ *A*_*i*_
11: $SK_{i}[a]\leftarrow g^{(r_{i}+H(a))\beta }$
12: **return** *SK*_*i*_
13: **end procedure**
14: **procedure** Encryption($M,g,g^{\beta },H,T$)
15: Choose a random element *s* from $Z_{p}$ and compute *C*_0_ as the product of *M* and *e*(*g*, *g*)^*αs*^.
16: For each $a_{j}\in T$, compute *C*_*j*_ = *g*^*s*^ ⋅ *H*(*a*_*j*_)^*s*^
17: **return** $\left(C_{0},{\left\{C_{j}\right\}}_{j\in T}\right)$
18: **end procedure**
19: **procedure** Decryption($C_{0},\left\{C_{j}\right\},SK_{i},T,H$)
20: Validate that *SK*_*i*_ satisfies T
21: Compute *e*(*g*^*β*^, *C*_*j*_)/*e*(*SK*_*i*_[*a*_*j*_], *g*) for each $a_{j}\in T$
22: Recover *M* from *C*_0_ using the computed values
23: **return** *M*
24: **end procedure**
25: **procedure** Challenge($g,g^{\alpha },g^{\beta },H,T,A$)
26: Choose random messages *M*_0_, *M*_1_, compute C* = Encryption(*M*_*b*_, …) for a random bit *b*
27: Adversary A attempts to guess *b* given C* and access to a decryption database.
28: **return** success if A guesses *b*, otherwise failure
29: **end procedure**

### 6.1 System setup initialization

The initial step involves invoking the setup function to generate the Primary key (PK) and Master key (MK). This involves choosing two bilinear groups, denoted as *G*_*T*_ and *G* each associated with a prime number *p*. We establish a bilinear map *e* : *G* × *G* → *G*_*T*_ based on two generators *g* and *v*. We define *U* = {addrID_*ui*_ ∣ 1 ≤ *i* ≤ *n*} as the user set and attr = {*a*_*j*_ ∣ 1 ≤ *i* ≤ *m*} as the global attribute set. A query list *v* initiates with the state, comprising a random parameter and an addrID. We select a hash function *H* : {0, 1}* → *G* and choose random elements $a,b\in Z_{p}$. The system ultimately outputs the Primary key PK and Master key MK, which are defined as follows:

$$\begin{array}{c} PK=\{g,v,h=v^{b},\hat{h}=g^{b},y=e{\left(g,v\right)}^{a},H\}.\\ MK=\{a,b\} \end{array}$$

### 6.2 Generate keys

To generate user-specific keys, KeyGen(MK, *L*_*ui*_, addrID_*ui*_) outputs (*DK*_*ui*_, *SK*_*ui*_). For each user *ui*, based on their attribute set *L*_*ui*_ and identification addrID_*ui*_, A random element *t* from the set of $Z_{p}$ is chosen, and the following calculations are performed:

$$\begin{array}{cc} D_{ui}^{\left(1\right)} & =g^{\left(a+t)/(b+bt\right)},\\ D_{ui}^{\left(2\right)} & ={\left\{g^{t}H\left(a_{j}\right)\right\}}_{a_{j}\in L_{ui}},\\ D_{ui}^{\left(3\right)} & =g^{{\text{addrID}}_{ui}bt},\\ D_{ui}^{\left(4\right)} & =h^{t}. \end{array}$$

These parameters, along with *t*, are recorded in *W*. The Secure Key $SK_{ui}=\left(D_{ui}^{\left(3\right)},D_{ui}^{\left(4\right)}\right)$ and the Decryption Key $DK_{ui}=\left(t,D_{ui}^{\left(1\right)},D_{ui}^{\left(2\right)}\right)$ are securely transmitted to user *ui*.

### 6.3 Data encryption

When medical practitioners create medical records, they invoke Encrypt(PK, Pi, Mi, R) to encrypt the data *M*_*i*_ for patient *u*_*i*_ under an access policy *P*_*i*_ and a revocation list *R*. The encryption process involves the following steps:

1. **Develop Access Tree Structure**: Develop an access structure *T*_*i*_ that corresponds to the stipulated policy *P*_*i*_. This structure can be defined using logical operators (AND, OR) and threshold gates to represent the necessary conditions for access.
2. **Random Element Selection**: Choose a random element *g* from the field $Z_{p}$ and perform the following calculations:
  $$\begin{array}{cc} C_{p}^{\left(1\right)} & =M_{i}\cdot e{\left(g,v\right)}^{ag}\\ C_{p}^{\left(2\right)} & =h^{g} \end{array}$$
3. **Secret Sharing**: Assign secret shares to the tree nodes using threshold secret sharing:
  - For an AND gate, distribute *g* among child nodes as per the (*t*, *n*)-threshold scheme.
  - For an OR gate and threshold gates, similarly distribute *g* using polynomials defined for the (*t*, *n*)-threshold.
4. **Leaf Node Calculation**: For every leaf *a*_*j*_ in *T*, compute
  $$C_{aj,k}^{\left(3\right)}=v^{g^{k}}H{\left(a_{j}\right)}^{-1},$$
  with *k* denoting the node index.
5. **Ciphertext Generation**: For each user in the revocation list R, generate a unique random number and compute the corresponding components of the ciphertext. The resulting ciphertext *C*_*pi*_ is defined as:
  $$C_{pi}=(C_{p}^{\left(1\right)},C_{p}^{\left(2\right)},{\left\{C_{aj,k}^{\left(3\right)}\right\}}_{a_{j}\in T_{i}},{\left\{C_{uj}^{\left(4\right)}\right\}}_{u_{j}\in R},{\left\{C_{uj}^{\left(5\right)}\right\}}_{u_{j}\in R}).$$

Smart contracts play a crucial role in this process by verifying the access policy Pi and ensuring that the encryption parameters are correctly calculated and securely stored. The smart contract automates the enforcement of access policies during the encryption process, ensuring that only authorized users can later decrypt the data.

### 6.4 Decryption delegation

The delegation function is defined as Delegate(PK, *L*_*ui*_, *DK*_*ui*_, $C_{p}^{\left(2\right)}$, $C_{j,i}^{\left(3\right)}$) which returns $\left(K_{ui},C_{i}^{\prime }\right)$. When a user requests access to their medical data, they send their attribute list *L*_*ui*_ and Decryption Key *DK*_*ui*_ to the AC. The Authentication center(AC) carries out the delegation process by identifying the minimal subset $L_{0}^{\prime }$ that fulfills the conditions of the access tree *T*_*i*_. For every attribute *a*_*j*_ within $L_{0}^{\prime }$, the AC then calculates the corresponding partial decryption factor:

$$\begin{array}{c} K_{ui}=\prod_{a_{j}\in L_{0}^{\prime }}e(D_{ui}^{\left(1\right)},C_{p}^{\left(2\right)})\cdot e{\left(D_{i}^{\left(2\right)},C_{a_{j},i}^{\left(3\right)}\right)}^{\lambda_{i}^{\left(0\right)}} \end{array}$$ (1)

where $\lambda_{i}^{\left(0\right)}$ are the Lagrange coefficients for the minimal set $L_{0}^{\prime }$. The AC then sends the transformed ciphertext $C_{i}^{\prime }$ along with *K*_*ui*_ to the user.

### 6.5 Data decryption

The decryption function Decrypt($C_{pi}^{\prime }$, *K*_*ui*_, *SK*_*ui*_) outputs $M_{i}^{\prime }$. Upon receiving $\left(K_{ui},C_{i}^{\prime }\right)$, the user can proceed with decryption if addrID_*ui*_ ∉ *R* and addrID_*ui*_ ∈ *S*. The system calculates the decryption key for each attribute $a_{j}\in L_{0}^{\prime }$ as follows:

$$\begin{array}{c} K^{\prime }=\prod_{j=1}^{r}{\left(\frac{e(D_{uj}^{\left(3\right)},C_{uj}^{\left(4\right)})}{e(C_{uj}^{\left(5\right)},D_{uj}^{\left(4\right)})}\right)}^{\frac{1}{{\text{addrID}}_{ui}-{\text{addrID}}_{j}}} \end{array}$$ (2)

where *r* is the number of attributes *a*_*j*_ where addrID_*uj*_ ∈ *R*. The final decryption key *K* and the original message $M_{i}^{\prime }$ are obtained by:

$$\begin{array}{c} K=\frac{K_{ui}}{K^{\prime }} \end{array}$$ (3)

$$\begin{array}{c} M_{i}^{\prime }=f(\frac{C_{p}^{\left(1\right)}}{K})=f\left(M_{i}\right) \end{array}$$ (4)

where *f* is a function that extracts the plaintext from the decrypted message. Only the intended user *ui* with addrID_*ui*_ ∈ *S* can recover the plaintext by applying the decryption function to the received $\left(K_{ui},C_{i}^{\prime }\right)$ using their secret key *SK*_*ui*_.

### 6.6 Monitoring of malicious users

The tracking function Trace(PK, *L*_*ui*_, *DK*_*ui*_) outputs either the user’s address identifier addrID_*i*_ or invalid. The Service management system (SMS) verifies *DK*_*ui*_ by checking if addrID can be located in list *W*, using parameter *t* to monitor decryption activities and provide referential data for tracking potentially malicious users. The process is bifurcated into two parts:

**Verify phase:** Given *L*_*ui*_, PK, and *DK*_*ui*_, the SMS computes:

$$\begin{array}{c} Rs_{1}=e\left(D^{\left(1\right)},h\right),\;Rs_{2}=y^{\delta }\cdot \prod_{a_{i}\in L_{ui}}e\left(D^{\left(2\right)},h_{2}\right)\cdot v^{H{\left(a_{i}\right)}^{-1}} \end{array}$$ (5)

If *Rs*_1_ = *Rs*_2_, the user passes verification as a legitimate user.

**Query phase.** If verification succeeds and *DK*_*ui*_ is valid, addrID is retrievable from list *W* using *t*, and the corresponding addrID is output. If verification fails, the output is false or invalid.

### 6.7 Data re-encryption

The encryption function Encrypt(PK, Π, *M*_*i*_, *R*_0_) yields $C_{pi}^{\prime }$. When the revocation list *R* changes to *R*_0_, only elements $\left\{C_{uj}^{\left(4\right)}\right\}$ for *uj* ∈ *R*_0_ and $\left\{C_{uj}^{\left(5\right)}\right\}$ for *uj* ∈ *R*_0_ in *C*_*pi*_ need updating. For instance, upon adding a malevolent user’s addrID_*e*_ to *R*, the new components $C_{e}^{\left(4\right)}=h^{\text{t}e}$ and $C_{e}^{\left(5\right)}=h^{{\text{addrID}}_{e}\cdot {\text{t}}^{-1}}$ are included in the updated sets. The revised ciphertext $C_{pi}^{\prime \prime }$ is defined as:

$$\begin{array}{c} C_{pi}^{\prime \prime }=\left(C_{p}^{\left(1\right)},C_{p}^{\left(2\right)},{\left\{C_{a_{j},k}^{\left(3\right)}\right\}}_{a_{j}\in T_{i}},{\left\{C_{uj}^{\left(4\right)}\right\}}_{uj\in R_{0}},{\left\{C_{uj}^{\left(5\right)}\right\}}_{uj\in R_{0}}\right) \end{array}$$ (6)

## 7 Security model

In this section, we outline the IND-CPA (Indistinguishability under Chosen Plaintext Attack) cryptographic security model. The model involves a protocol between an adversary, denoted as A, and a challenger, denoted as B. The steps of the protocol are as follows:

1. **Initial Step:** The opponent A selects a specific access structure T* and compiles a group of revoked users R*. This data is then passed to the challenger B.
2. **Setup Phase:** The challenger B generates a *MK* and a *PK*. The PK is shared with A, while the MK is kept secret.
3. **Phase 1:** A requests multiple secret keys *SK*_*u*_ that correspond to a predefined list L*. B produces these keys using a designated key generation algorithm.
4. **Challenge Step:** A presents two messages of equal size, *M*_1_ and *M*_2_, to B. B chooses a random bit *u* from {0, 1} and encrypts one of the messages using T* and R*. The ciphertext *C*_*p*_ is then given to A.
5. **Phase 2:** This phase is similar to the First Phase, with A continuing to request secret keys.
6. **Prediction Step:** A predicts the value of *u* as *u*′. The adversary’s success is defined if *u*′ = *u*, with the probability given by $\text{Pr}\left[u^{\prime }=u\right]-\frac{1}{2}$.

The security of our model is considered robust if adversaries constrained to polynomial time have but a negligible success probability in the aforementioned game, thereby affirming the system’s resilience against plain text disclosure attacks.

## 8 Security proof

Consider a scenario where an adversary, denoted as R, successfully compromises the robustness of an encryption protocol within the IND-CPA security. In response, we formulate an algorithm, A, that utilizes R to solve the DBDH problem. This is achieved by verifying whether *e*(*g*, *g*)^*z*^ is identical to *e*(*g*, *g*)^*abc*^.

The process is structured as follows:

1. **Initial Step:** Adversary R picks a specific access structure, $T^{\prime }$, and a group of revoked users, $R^{\prime }$. These selections are forwarded to the challenger, A.
2. **Preparation Phase:** A begins by executing the initial setup algorithm. It chooses a random element *x* from $Z_{p}$ and calculates *a* as *ab*+*x*, which leads to *y* = *e*(*g*, *g*)^*a*^. Subsequently, A generates a series of random elements ${\left\{b_{i}\right\}}_{i=1}^{r}$, ensuring their collective sum is *b*, and computes $h=\prod_{i=1}^{r}g^{b_{i}}$. This results in the creation of both the public key PK and the master key MK, with PK being revealed to R.
3. **Phase 1:** Here, R requests private keys for users who are neither part of $T^{\prime }$ nor among the revoked. In response, A generates and provides the necessary keys to R.
4. **Challenge Stage:** R proposes two messages of equal length, *M*_1_ and *M*_2_. A randomly chooses *u* from {0, 1} and encrypts *M*_*u*_ considering both $T^{\prime }$ and $R^{\prime }$, using a random *s*. The resultant ciphertext, $C_{P}^{\prime }$, is then handed over to R.
5. **Phase 2:** Similar like First Phase, R continues to make further private key requests, to which A responds as required.
6. **Concluding Guess:** R then attempts to deduce *u* and presents its guess as *u*′. If *u*′ matches *u*, A concludes that *e*(*g*, *g*)^*z*^ and *e*(*g*, *g*)^*abc*^ are equivalent. Otherwise, A infers that *e*(*g*, *g*)^*z*^ is a random value.

## 9 Security analysis of the proposed method

The security of electronic health records (EHRs) in cloud environments is of paramount importance. Our proposed algorithm integrates blockchain and smart contracts with cloud-based healthcare Service Management Systems (SMS) to ensure robust security measures. This section presents a detailed security analysis, demonstrating the resilience of our scheme against various cyber threats.

### 9.1 Data confidentiality

Data confidentiality ensures that sensitive information is accessible only to authorized users. In our system, we achieve confidentiality through the use of Ciphertext-Policy Attribute-Based Encryption (CP-ABE). CP-ABE embeds access control policies within the ciphertext, ensuring that only users whose attributes satisfy the policy can decrypt the data. This method is particularly effective in preventing unauthorized access to EHRs.

### 9.2 Data integrity

Data integrity involves maintaining the accuracy and consistency of data over its lifecycle. Blockchain technology inherently provides immutability and transparency, ensuring that once data is recorded, it cannot be altered or tampered with. Each transaction involving EHRs is hashed and stored on the blockchain, and any attempt to alter the data can be easily detected. This guarantees that the data remains intact and unaltered.

### 9.3 Authentication and authorization

Our system employs a dual authentication mechanism to enhance security. Patients and medical practitioners are required to authenticate their identity using a combination of public keys (PK) and master keys (MK). The authentication center (AC) validates user identities before granting access to EHRs. This two-factor authentication mechanism ensures that only legitimate users can access the data.

### 9.4 Access control and revocation

The integration of CP-ABE with blockchain allows for fine-grained access control. Access policies are dynamically embedded within the encrypted data, allowing only users with matching attributes to decrypt it. Furthermore, our system supports direct access revocation, where the SMS can revoke data access in case of suspicious activities. The blockchain ledger maintains a log of all access attempts, making it easier to track and revoke permissions for malicious users.

### 9.5 Data auditing and traceability

Blockchain’s decentralized ledger provides a transparent and immutable audit trail for all data transactions. This audit trail is crucial for tracking data access and modifications. Smart contracts within the system automate the logging of all access attempts, ensuring that any unauthorized access is promptly detected and logged. This feature enhances the traceability of data and simplifies auditing processes.

### 9.6 Resistance to cyber attacks

Our system effectively handles several critical security threats through its robust design. The dual authentication mechanism and CP-ABE encryption ensure strong defenses against impersonation and dictionary attacks by requiring both public and master keys for access. The decentralized nature of blockchain, coupled with consensus mechanisms, mitigates the risks of Sybil and 51% attacks, while multiple peer connections protect against Eclipse attacks. Time-stamped transactions and nonce values prevent replay attacks by ensuring the uniqueness of each transaction. Additionally, the transparent audit trail provided by the blockchain helps detect and prevent insider threats, ensuring comprehensive security for electronic health records (EHRs).

## 10 Protocol design

### 10.1 Scheme overview

We introduce a suite of cryptographic operations aimed at enabling the traceable and secure management of CEMRs within cloud storage systems. These operations include:

- **IDGen**(***password***): This method generates a distinct account identifier, named **addrID**, based on the user’s password. When the AC confirms the policy through the **Delegate()** function, this method yields the decryption elements along with the encrypted data. The user can then apply the **Decrypt()** function for data decryption.
- **Setup**(***security_param***): This function takes a designated security parameter and establishes the system’s *PK* and the *MK*.
- **KeyGen**(***MK, attributes, addrID_ui***): Generates a user-specific decryption key *DK*_*ui* and secret key *SK*_*ui*, utilizing the master key *MK*, user’s attributes, and account identifier.
- **Encrypt**(***PK, policy, M, R***): Encrypts a message *M* using the public key *PK*, an access policy *policy*, and a revocation directive *R*, producing an encrypted output *Cp*_*i*.
- **Delegate**(***PK, attributes, DK_ui, Cp_i***): Converts the ciphertext *Cp*_*i* into a form suitable for decryption *Cp*′_*i*, using the decryption key *DK*_*ui*, public key, and user attributes.
- **Decrypt**(***C’pi, K_ui, SK_ui***): Deciphers the transformed ciphertext *C*′*pi* back into the original message *M* using the secret key *SK*_*ui* and decryption parameter *K*_*ui*.
- **Trace**(***PK, attributes, DK_ui***): Identifies a user’s account *addrID*_*ui* or yields a placeholder based on the public key, user attributes, and decryption key.
- **ReEncrypt**(***PK, policy, M, R’***): Re-encrypts a message in accordance with an updated policy or revocation list, generating a fresh ciphertext *Cp*′_*i*.
- **TransactionSave**(***privateKey, addrID, content, timestamp***): Logs transactions on the blockchain using the sender’s identifier *addrID*, transaction content *content*, and the timestamp *timestamp*, with the private key *privateKey* serving for authentication. It confirms whether the transaction was successful.

The system’s operational process is as follows:

1. Users create a blockchain account, which in turn generates an address *addrID*_*ui*_.
2. The SMS implements the Initialization Procedure to set up the system’s PK and MK, ensuring they conform to the defined attribute standards of the system.
3. Medical practitioners draft access policies in consideration of patient privacy and encrypt relevant data using **Encrypt()** before dispatching it to the CSP.
4. Users request access to medical data from the CSP by submitting their *DK*_*ui*_ and attribute set.

## 11 Simulation experiment

This part of the document evaluates the performance of the suggested framework. Performance metrics were gathered by conducting 700 iterations of linear pairing and exponentiation calculations on a system equipped with an Intel Core i7 processor clocked at 2.7 GHz, running a 64-bit version of Windows 10, and 32 GB of RAM. The selection of Ethereum(Ganache) and Truffle Node for the data environment and for implementation was motivated by its simplified verification process.

In evaluating the performance of networked systems, particularly those based on blockchain technology, throughput and delay serve as crucial metrics. Throughput is the measure of how many transactions a system can handle per second, while delay denotes the time taken for data to be transmitted between nodes, often described as latency. We assessed the system’s read and write performance by varying the transaction load from 100 to 700 transactions per second as shown in Table 3.

**Table 3:** Selected transaction data.

Active Transactions	Transaction Time (ms)	Throughput (Tx/s)
100	2167	46.1
200	3202	62.4
300	4305	69.6
400	6462	61.9
500	6895	72.5
600	8426	71.2
700	10158	68.9

**Fig 3:**
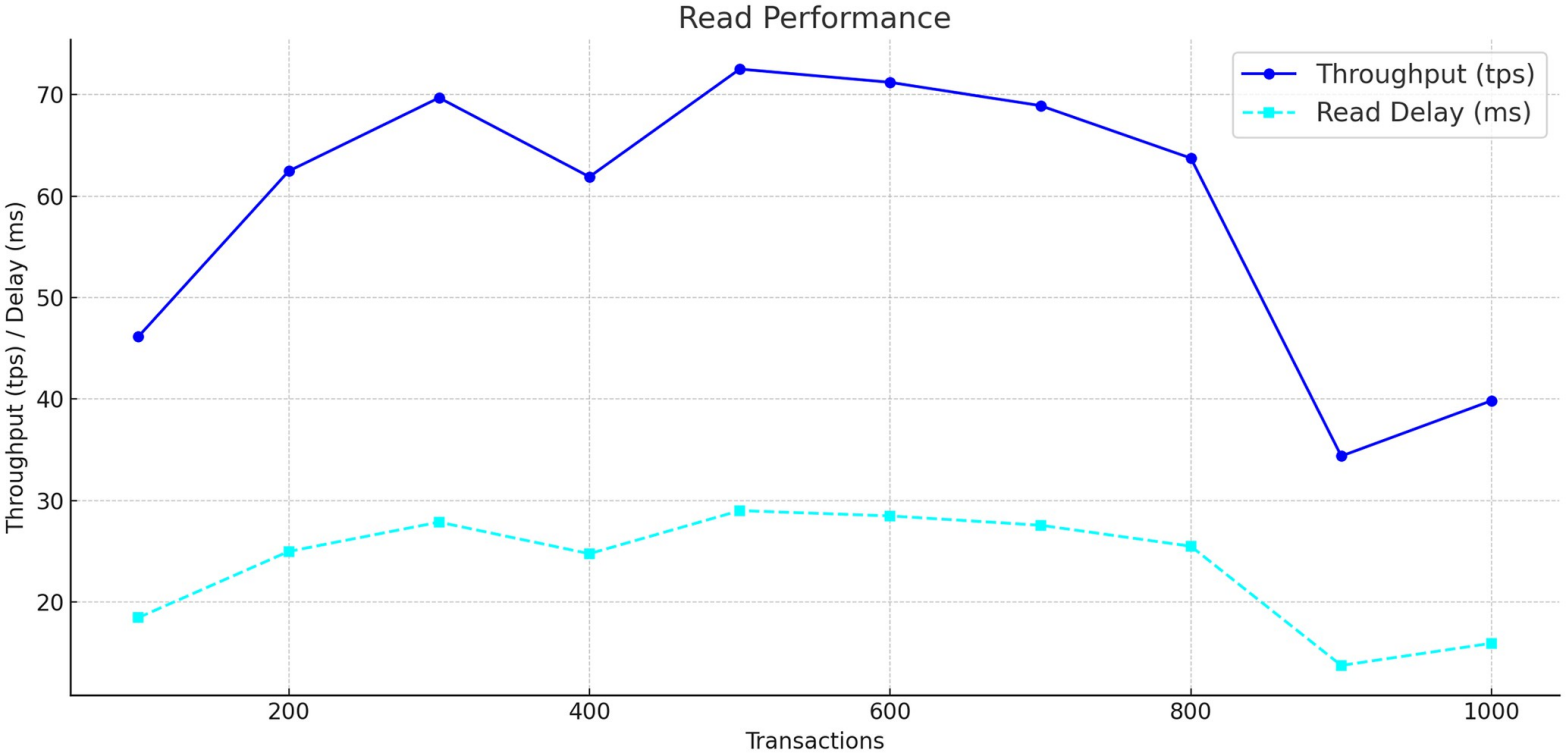
Read performance and throughput.

**Fig 4:**
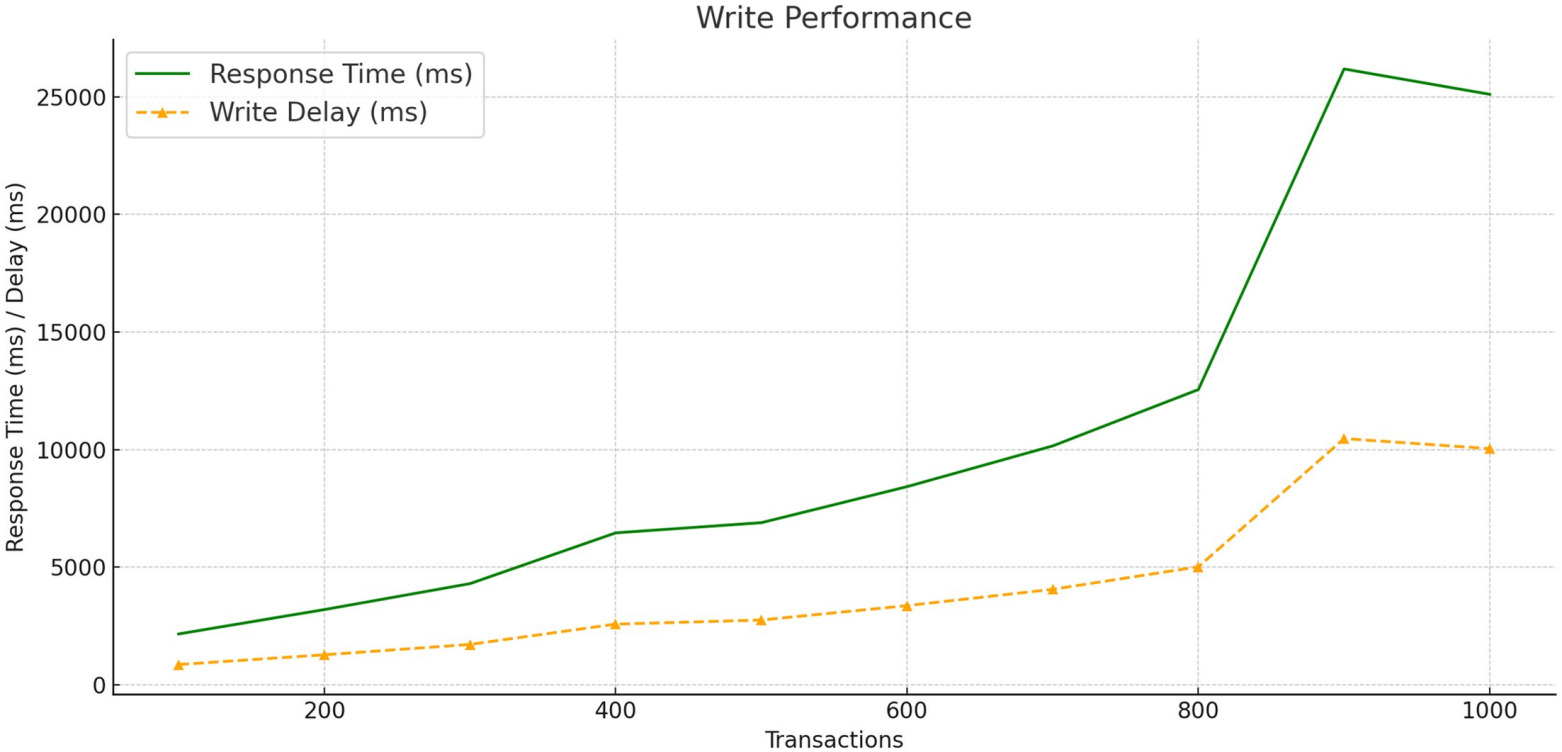
Write performance and throughput.

The resulting graphs Figs 3 and 4 show the read and write performances. In the read performance graph, throughput is represented by the blue line, which shows an increase with the number of transactions. The cyan dashed line indicates the read delay, showing a trend that suggests an increase in latency as transaction volume rises. For write performance, represented by the green line, throughput initially increases with the number of transactions, peaks, and then slightly decreases. The orange dashed line reflects write delay, illustrating a rise in latency with increased transaction volume.

**Fig 5:**
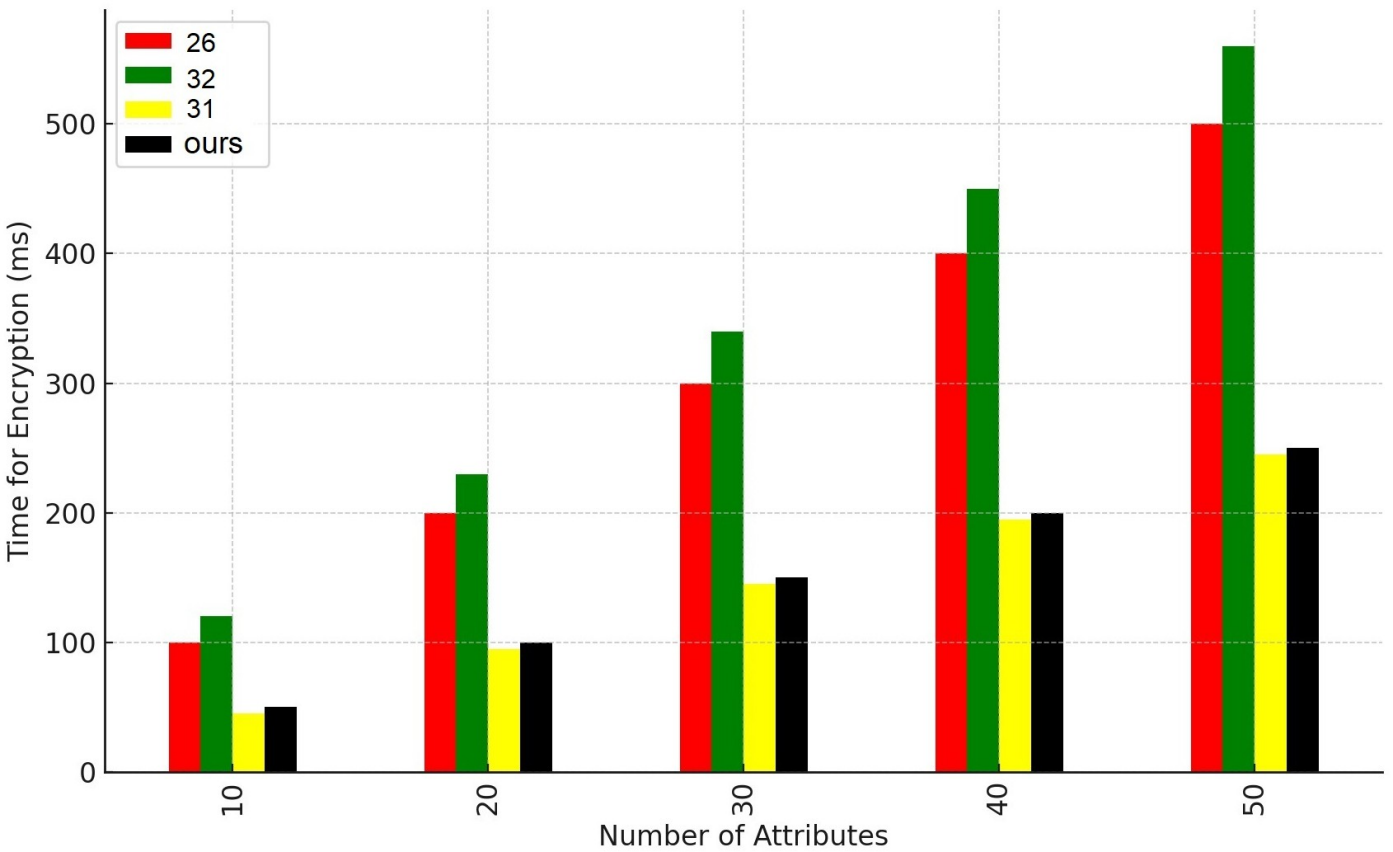
Encryption performance.

**Fig 6:**
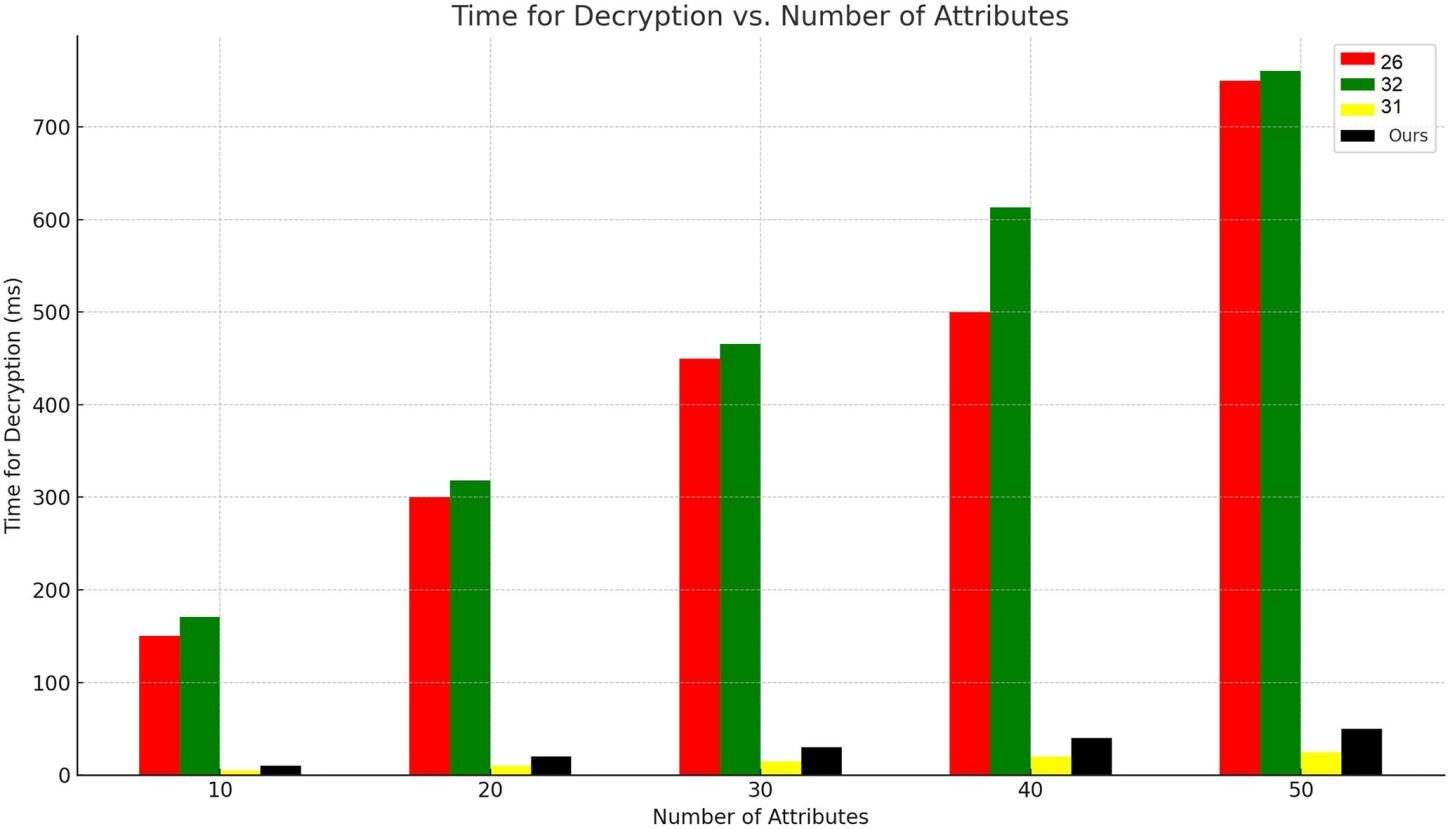
Decryption performance.

**Fig 7:**
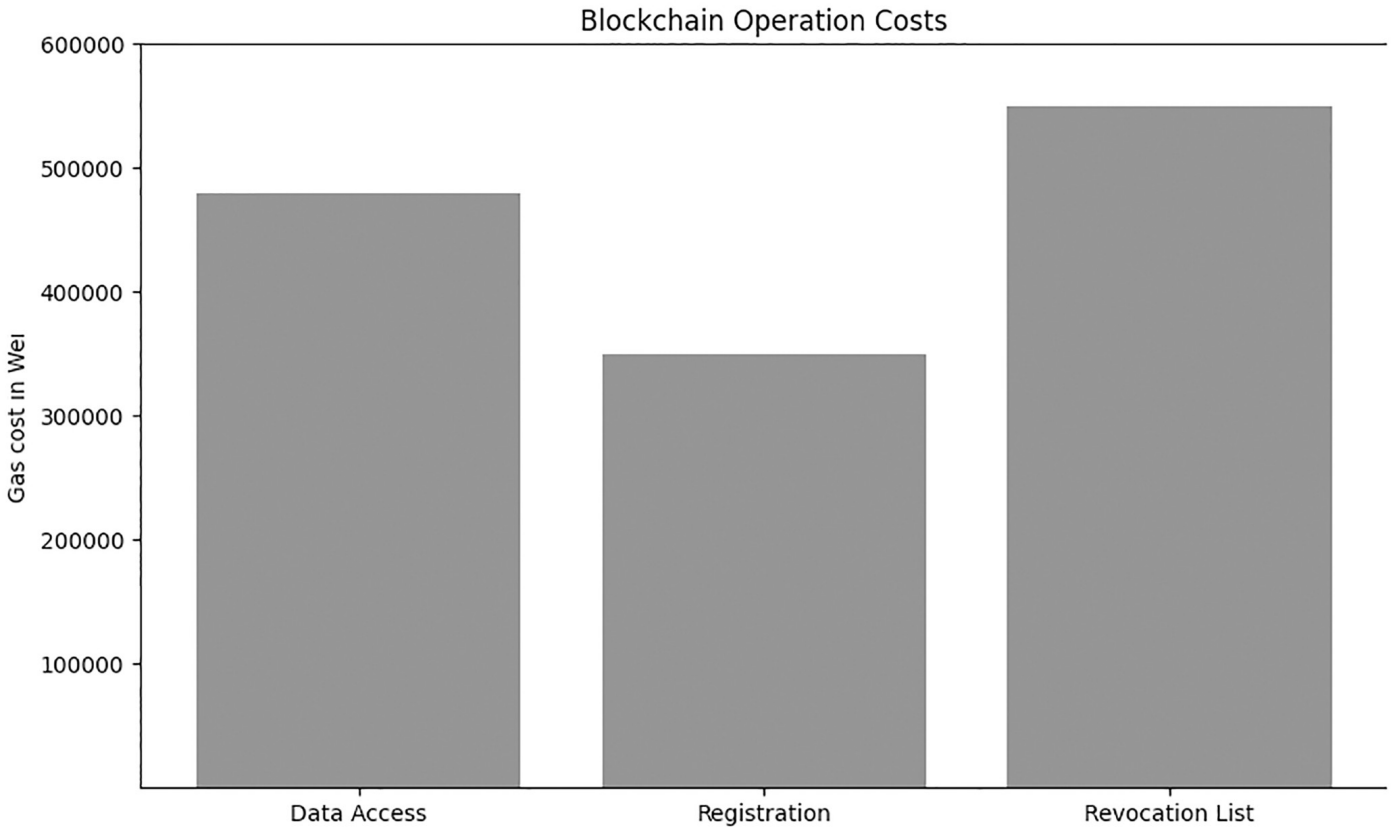
Smart contract cost in wei.

On the other hand The comparison results for time overhead between our proposal and other works [26, 31, 32] are shown in Figs 5 and 6 which shows the system performance in terms of encryption and decryption times. Notably, both the encryption and decryption times exhibit an upward trend as the number of transactions increases but still our system performance is better then other compared systems. Fig 7 shows our smart contract cost in wei which we have implemented by using solidity truffle environment and ganache Ethereum simulator.

These graphs show that our blockchain system offers higher throughput and lower latency during read operations compared to write operations. The read operations are handled efficiently by the system, while the write operations introduce more latency, likely due to the complexities of data validation and recording on the blockchain and CSP. The overall results point to a blockchain solution that is effective in managing high throughput and maintaining low latency, which aligns with the operational requirements of hospital data management systems.

## 12 Comparison

Our work introduce an integrated security framework that significantly enhances the protection and accessibility of Electronic Health Records (EHRs) through the synergy of blockchain, smart contract and cloud computing technologies. This work transcends previous research by delivering a more scalable, efficient, and practical solution for healthcare data management, incorporating advanced traceability and direct access revocation mechanisms. The proposed model not only addresses current challenges in data security and privacy but also demonstrates a clear, implementable pathway towards improving healthcare information systems, marking a pivotal advancement in secure digital health records management, as shown in Table 4.

**Table 4:** Comparison of our system with others.

Functionalities	Our work	[31]	[32]	[26]
Scalability	Yes	Yes	No	Yes
Blockchain Utilization	Yes	No	Yes	No
Data Traceability	Yes	Yes	Yes	No
Data Auditing and Provenance	Yes	No	Yes	No
Identity Management	Yes	Yes	Yes	Yes
Immutability	Yes	No	Yes	No
User Authentication	Yes	Yes	Yes	Yes

## 13 Future works

In future work, we aim to explore the integration of advanced machine learning algorithms with our blockchain-cloud-based framework to enhance the predictive analysis and personalization of healthcare services. This will involve developing AI-driven models for real-time health data analysis, leading to more accurate and timely disease diagnosis and treatment recommendations. Additionally, we plan to expand the scope of our platform to include interoperability with a wider range of electronic health record systems and medical devices, ensuring seamless data exchange across different healthcare providers and platforms. Another key area of focus will be enhancing the security features of our system by incorporating newer cryptographic techniques and exploring quantum-resistant algorithms to safeguard against evolving cyber threats. Furthermore, we intend to conduct extensive field trials to validate the efficacy of our proposed system in diverse healthcare settings, ranging from urban hospitals to remote healthcare units, thus broadening its applicability and impact.

## 14 Conclusion

It is a huge challenge to take secure storage and sharing of Medical data among different cloud-based hospital systems. We proposed a robust algorithm that integrates blockchain technology and smart contracts with cloud computing to ensure secure and authenticated access to health data. The proposed solution addresses critical concerns in the healthcare sector, including data privacy, security, and integrity, by leveraging the decentralized nature of blockchain and the scalability of cloud computing. The results demonstrate that this integrated approach not only enhances the security of sensitive health data but also ensures seamless and efficient data access for authorized users. This algorithm sets a new standard in healthcare data management, offering a promising path forward for healthcare providers and patients alike in the age of digital health information.
